# P-1195. Activity of manogepix against surveillance yeast isolates collected from the United States in 2023

**DOI:** 10.1093/ofid/ofaf695.1388

**Published:** 2026-01-11

**Authors:** Marisa Winkler, Samuel Edeker, Abby Klauer, Paul Rhomberg, Mariana Castanheira

**Affiliations:** Element Materials Technology/Jones Microbiology Institute, North Liberty, Iowa; Element Materials Technology/Jones Microbiology Institute, North Liberty, Iowa; JMI Laboratories, North Liberty, Iowa; Element Materials Technology/Jones Microbiology Institute, North Liberty, Iowa; Element, North Liberty, IA

## Abstract

**Background:**

Manogepix (MGX) targets the fungal Gwt1 enzyme. Fosmanogepix, the prodrug of MGX is undergoing phase 3 clinical trials for treating invasive candidiasis and mould infections. Here, we assessed the *in vitro* activity of MGX against yeast isolates collected from patients with invasive infections.

**Figure:**
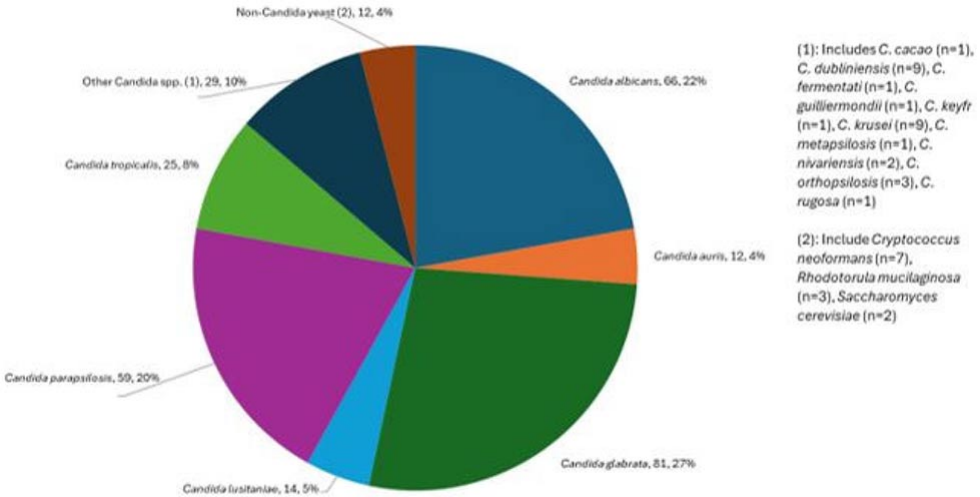
Tested yeast in United States manogepix surveillance program in 2023

**Figure:**
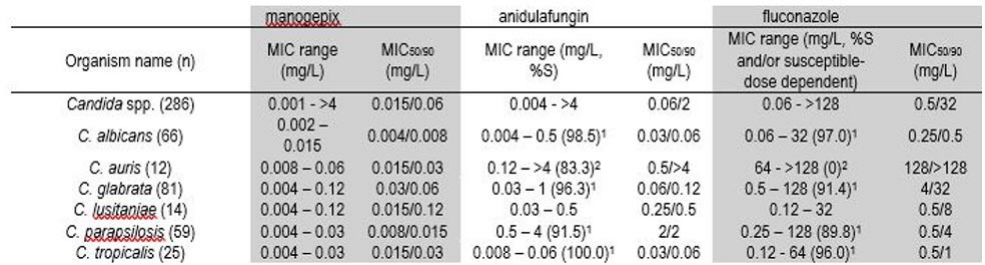
MIC50, MIC90, for manogepix and select comparators of organisms with more than 10 species tested 1 Based on breakpoints from CLSI M27M44S 3rd Ed (2022) 2 Based on CDC resistant-only breakpoints (https://www.cdc.gov/candida-auris/hcp/laboratories/antifungal-susceptibility-testing.html)

**Methods:**

A total of 298 yeast isolates were collected in 2023 from 24 hospitals in the United States (US). Isolates were tested by reference broth microdilution method according to CLSI guidelines against MGX, anidulafungin (AND) and fluconazole (FLC). Susceptible (S) or nonsusceptible (NS) isolates were interpreted by CLSI and CDC guidelines (resistant-only for *C. auris*). No breakpoints are available for MGX.

**Results:**

There were 19 different species of yeast represented (Figure 1). *Candida glabrata* was the most common organism (81/298, 27.2%), followed by *C. albicans* (66/298, 22.1%), *C. parapsilosis* (59/298, 19.8%), and *C. tropicalis* (25/298, 8.4%). There were 12 *C. auris* (4.0%). For non-*Candida* yeast, 3 different species were represented. MGX MIC_50/90_ values against *Candida* spp. were 0.015/0.06 mg/L (separated by species in Table 1). The MIC_50/90_ was lower for MGX than all other comparators. For *C. albicans*, 2 isolates were FLC-NS (one intermediate, I, one resistant, R) with MGX MICs of 0.008 mg/L; for the 1 isolate NS to AND, MGX MIC was 0.008 mg/L. Seven FLC-R and 3 AND-NS *C. glabrata* isolates exhibited MGX MICs ranging from 0.03 – 0.06 mg/L. MGX MICs ranged from 0.008 to 0.06 mg/L against *C. auris*; all isolates were FLC-R and 2 isolates were AND-R. Elevated MGX MICs (≥ 0.5 mg/L) were seen for *C. krusei* (9/9 isolates) and some *Cryptococcus neoformans* (2/7 isolates).

**Conclusion:**

MGX has potent *in vitro* activity among diverse yeast species collected in a US-based surveillance program. For most species, MIC_90_ was ≤ 0.03 mg/L including organisms of critical concern like *C. auris*. Elevated MICs were seen with *C. krusei* and some *Cryptococcus neoformans* which has also been observed previously. These data indicate that MGX is a promising novel antifungal agent for the treatment of infections due to yeast.

**Disclosures:**

Marisa Winkler, MD, PhD, Basilea: Advisor/Consultant|Basilea: Grant/Research Support|GSK: Advisor/Consultant|GSK: Grant/Research Support|Melinta Therapeutics: Advisor/Consultant|Melinta Therapeutics: Grant/Research Support|Mundipharma: Advisor/Consultant|Mundipharma: Grant/Research Support|Pfizer: Advisor/Consultant|Pfizer: Grant/Research Support|Pulmocide: Advisor/Consultant|Pulmocide: Grant/Research Support Mariana Castanheira, PhD, Melinta Therapeutics: Advisor/Consultant|Melinta Therapeutics: Grant/Research Support

